# Supplementary material for: Deconvolution of cell type-specific drug responses in human tumor tissue with single-cell RNA-seq
Source: Genome Med. 2021 May 11;13:82. doi: 10.1186/s13073-021-00894-y (PMC8114529; doi:10.1186/s13073-021-00894-y)
Supplement: Supplementary file 1 — Additional file 1: Supplementary Materials For: Deconvolution of Cell Type-Specific Drug Responses in Human Tumor Tissue with Single-Cell RNA-seq. This file includes supplementary figures: Fig. S1-S15. and supplementary tables: Table S1-S3. [file 13073_2021_894_MOESM1_ESM.pdf]

Supplementary Materials For: Deconvolution of Cell Type-Specific Drug Responses in Human Tumor Tissue with Single-Cell RNA-seq

Supplementary Figures

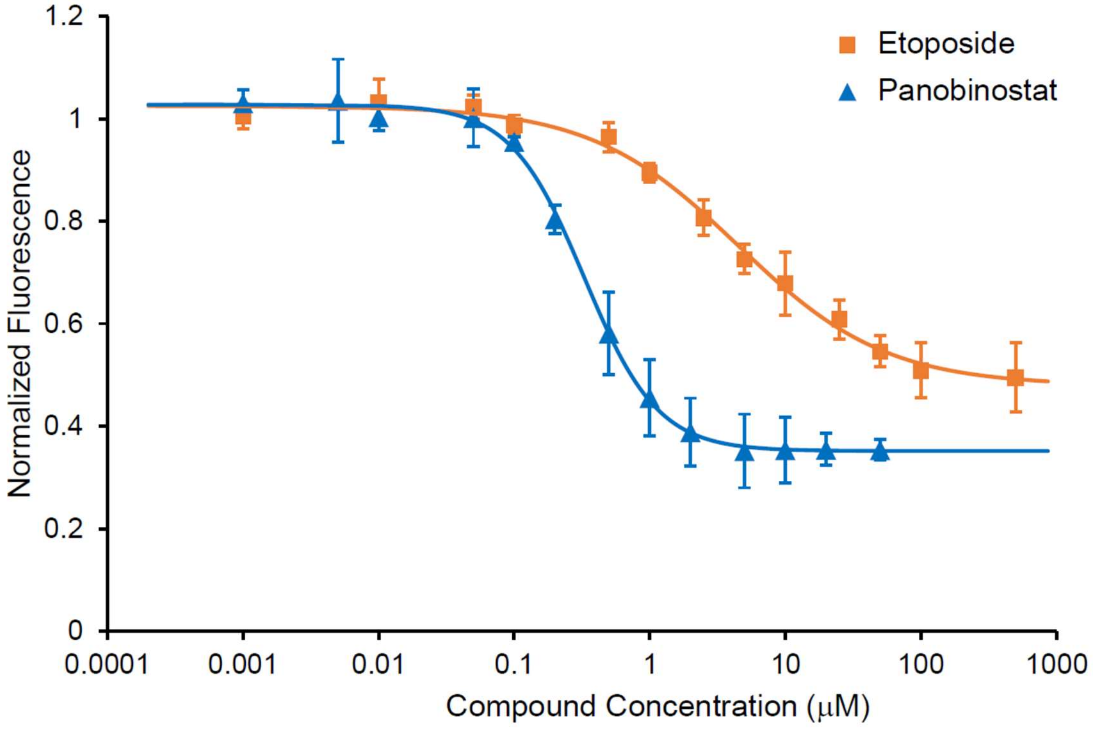

Fig. S1. Dose response curves for etoposide and panobinostat obtained for TS543 patient-derived glioma neurospheres. TS543 cells were cultured and maintained in the NeuroCult™ NS-A Basal Medium (Stem Cell Technologies) supplemented by 10% NeuroCult™ Proliferation Supplement (Stem Cell Technologies), 2mg/ml Heparin (Stem Cell Technologies), 10ng/ml bFGF (Stem Cell Technologies) and 20ng/ml EGF (Stem Cell Technologies) in a humidified incubator at 37°C and 5% CO_2. For the cell viability assay, TS543 cells were plated in a 96 well plate at 3200 cells/well and were cultured for 5 days to form cell spheres. Cell spheres were then exposed to various concentrations of Etoposide or Panobinostat or corresponding volume of vehicle (DMSO) for 48 hours followed by the cell viability detection using PrestoBlue® Reagent (Invitrogen™) as instructed by the manufacturer. To achieve a robust fluorescence readout, TS543 spheres were incubated with PrestoBlue® Reagent for 60 minutes in the humidified incubator at 37°C and 5% CO_2. Then the fluorescence was detected using excitation wavelength of 560 nm and emission of 590 nm on microplate reader (BioTek). To calculate the normalized fluorescence, fluorescence readout of each well was first subtracted by the mean fluorescence of blank wells to reduce background. Then we calculated the mean of background-subtracted fluorescence from technique repeats of each treatment condition. Finally, the compound-treated mean was divided by the corresponding vehicle-treated mean as the normalized fluorescence. Data represented in the figure showed the mean±SEM of normalized fluorescence from four independent experiments.

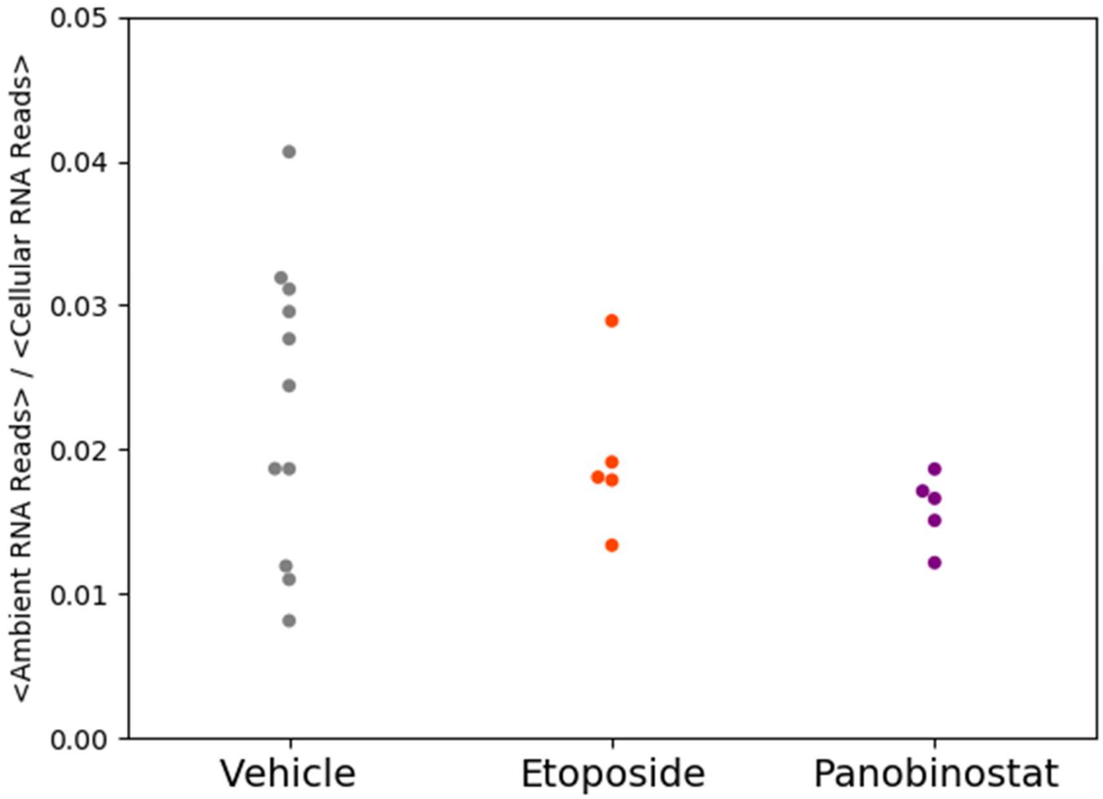

Fig. S2. Average number of reads per barcode for cell-identifying barcodes assigned to cells divided by the average number of reads per barcode for barcodes that were not assigned to cells (ambient RNA) based on the EmptyDrops algorithm for all vehicle-, etoposoide-, and panobinostat-treated slice cultures from PW029, PW030, PW032, PW034, PW036, and PW040. There are no significant differences in the apparent level of ambient RNA contamination between the drug- and vehicle-treated samples.

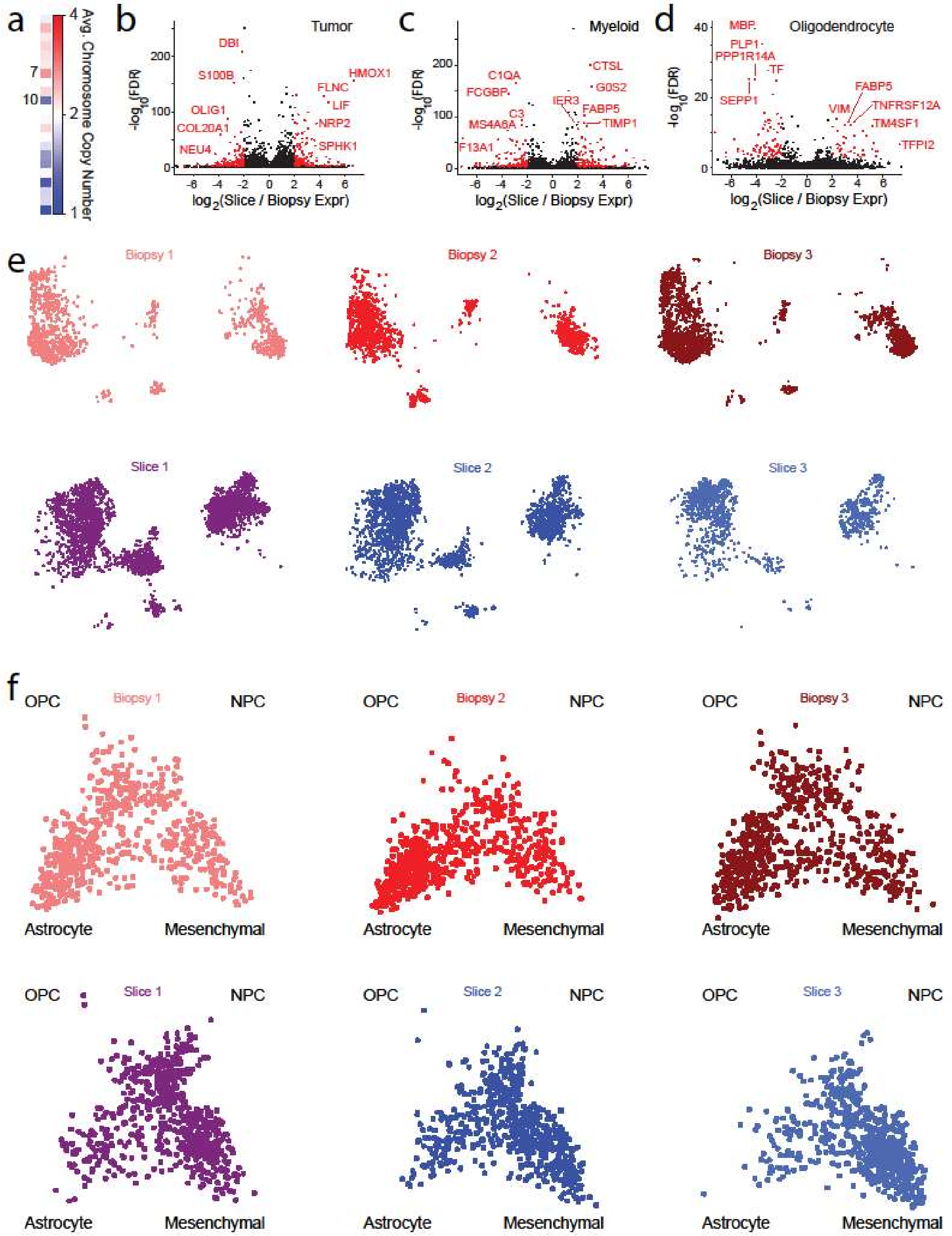

Fig. S3. **a** Heatmap showing the average chromosomal copy number from whole genome sequencing of PW032. **b** Volcano plot of differential expression analysis between slice culture and biopsy specimens for the transformed tumor cells in PW032. Genes highlighted in red have FDR<0.05 and absolute fold-change > 4. **c** Same as **b** for the myeloid cells in PW032. **d** Same as **b** for the oligodendrocytes. **e** UMAP embedding in **Fig. 1b** displayed for each sample. **f** Scatter plot of four-state model in **Fig. 1g** displayed for each sample.

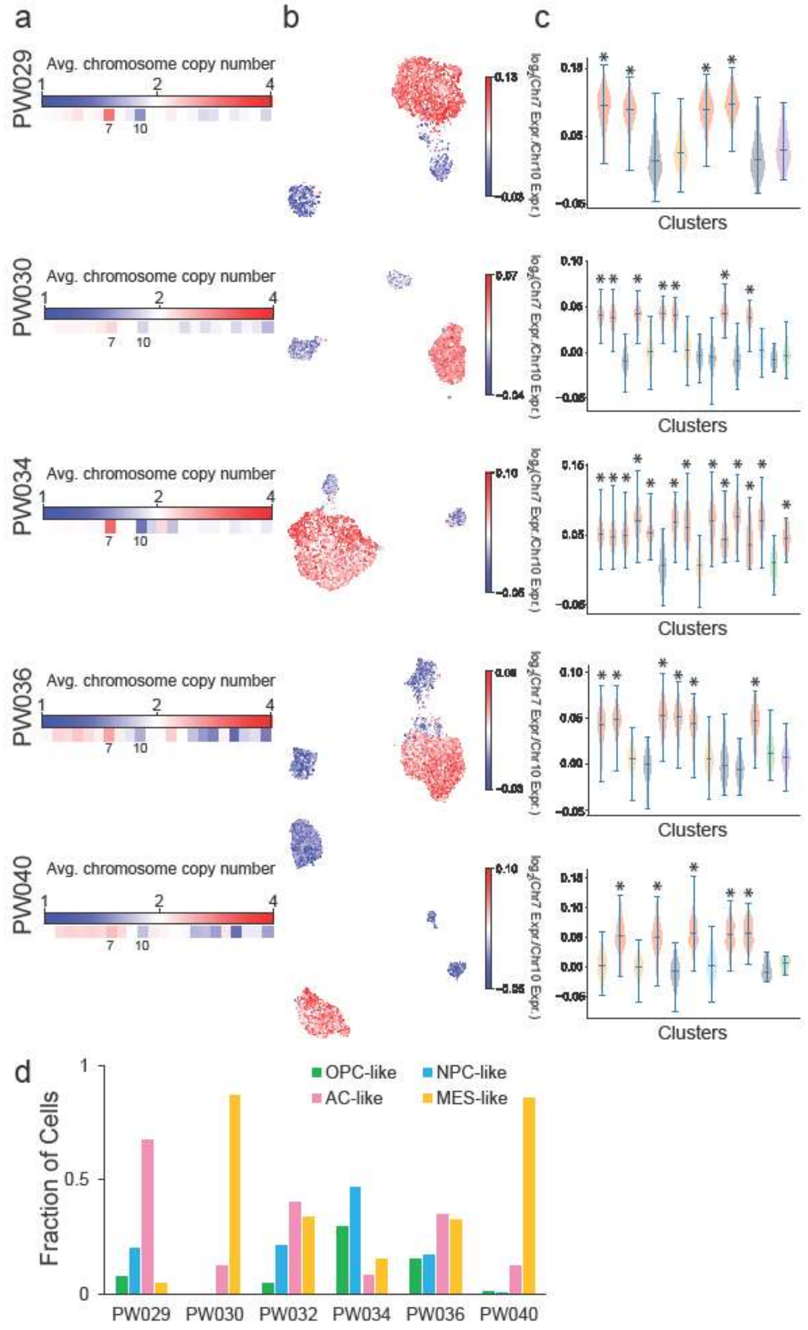

Fig. S4. **a** Heatmaps showing the average chromosomal copy number from whole genome sequencing of PW029, PW030, PW034, PW036, and PW040. **b** UMAP embeddings of scRNA-seq profiles from PW029, PW030, PW034, PW036, and PW040 slice cultures colored by the log-ratio of Chr. 7 to Chr. 10 average expression where a high ratio (red) indicates malignant transformation. **c** Violin plots showing the distributions of log-ratios of Chr. 7 to Chr. 10 average expression for each Phenograph cluster identified for PW029, PW030, PW034, PW036, and PW040 slice cultures. **d** Fractional abundance of each of the four major GBM transformed cell states from the two-dimensional projection in **Fig. 2b** for the transformed cells in the PW029, PW030, PW032, PW034, PW036, and PW040 vehicle-treated slice cultures.

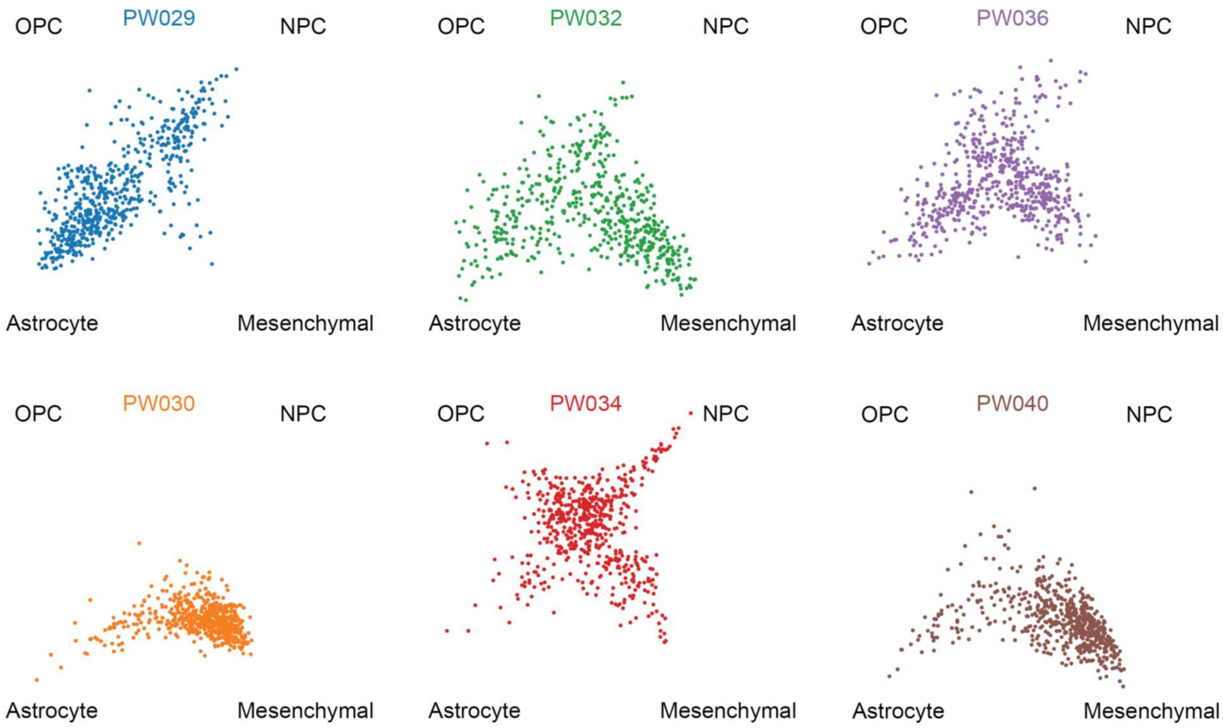

Fig. S5. Scatter plot of four-state model in **Fig. 2b** displayed for each sample.

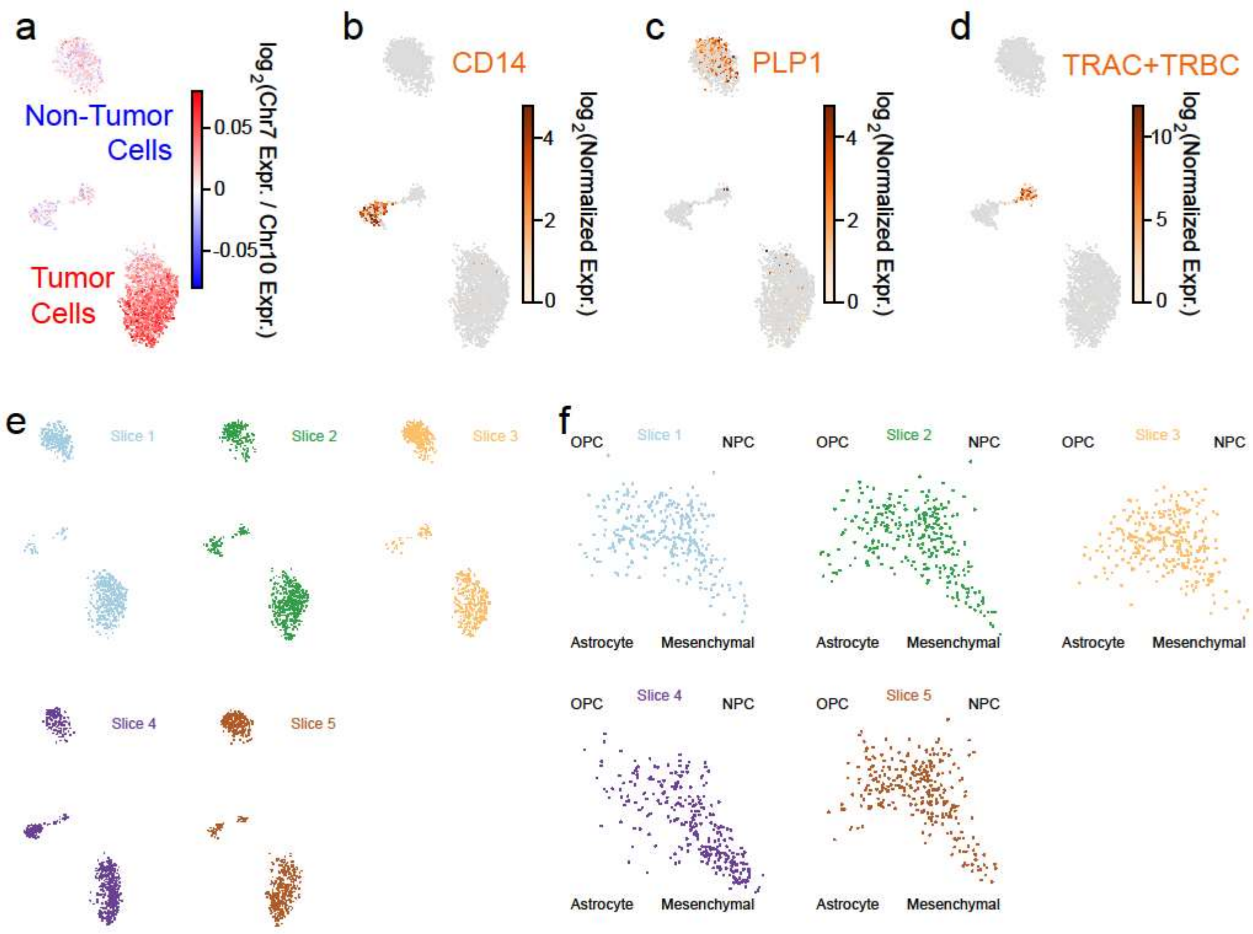

Fig. S6. **a** UMAP embedding of scRNA-seq profiles from five untreated slice cultures taken within 3.5 mm of each other from PW040 colored by the log-ratio of Chr. 7 to Chr. 10 average expression where a high ratio (red) indicates malignant transformation (same as UMAP in **Fig. 2c**). **b** Same as **a** colored by expression of the myeloid marker CD14. **c** Same as **a** colored by expression of the oligodendrocyte marker PLP1. **d** Same as **a** colored by total expression of the T cell receptor constant regions (TRAC, TRBC1, TRBC2). **e** UMAP embedding in **Fig. 2c** displayed for each sample. **f** Scatter plot of four-state model in **Fig. 2d** displayed for each sample.

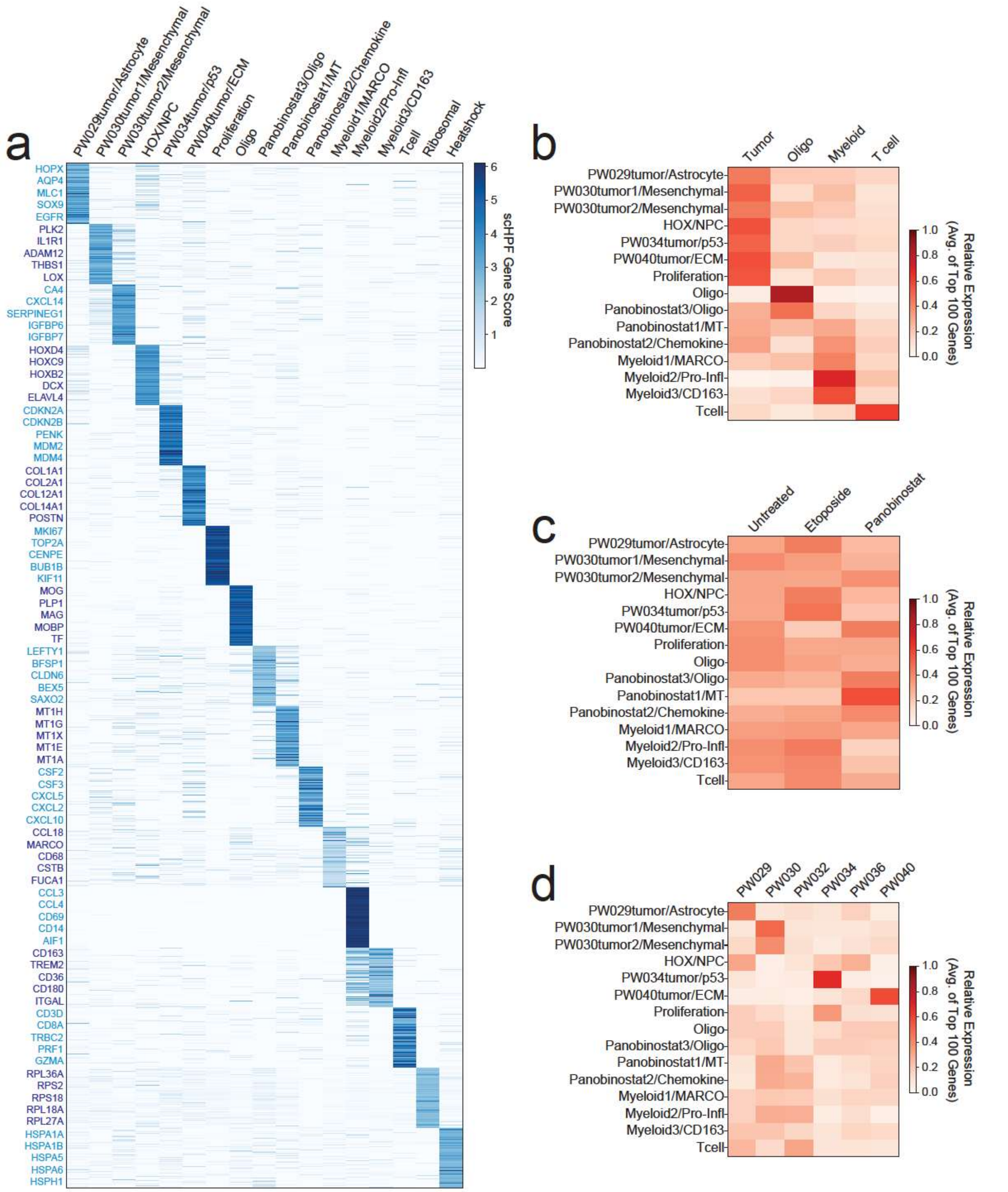

Fig. S7. **a** Heatmap showing the scHPF gene scores for representative high-scoring marker genes for each scHPF factor for the model in **Fig. 4**. **b** Heatmap showing the relative average expression of the top 100 markers in each scHPF factor for the transformed (tumor) cells, oligodendrocytes, myeloid cells, and T cells. **c** Same as **b** for all cells in the vehicle-, etoposide-, and panobinostat-treate slices. **d** Same as **b** for all cells from all slices for each patient.

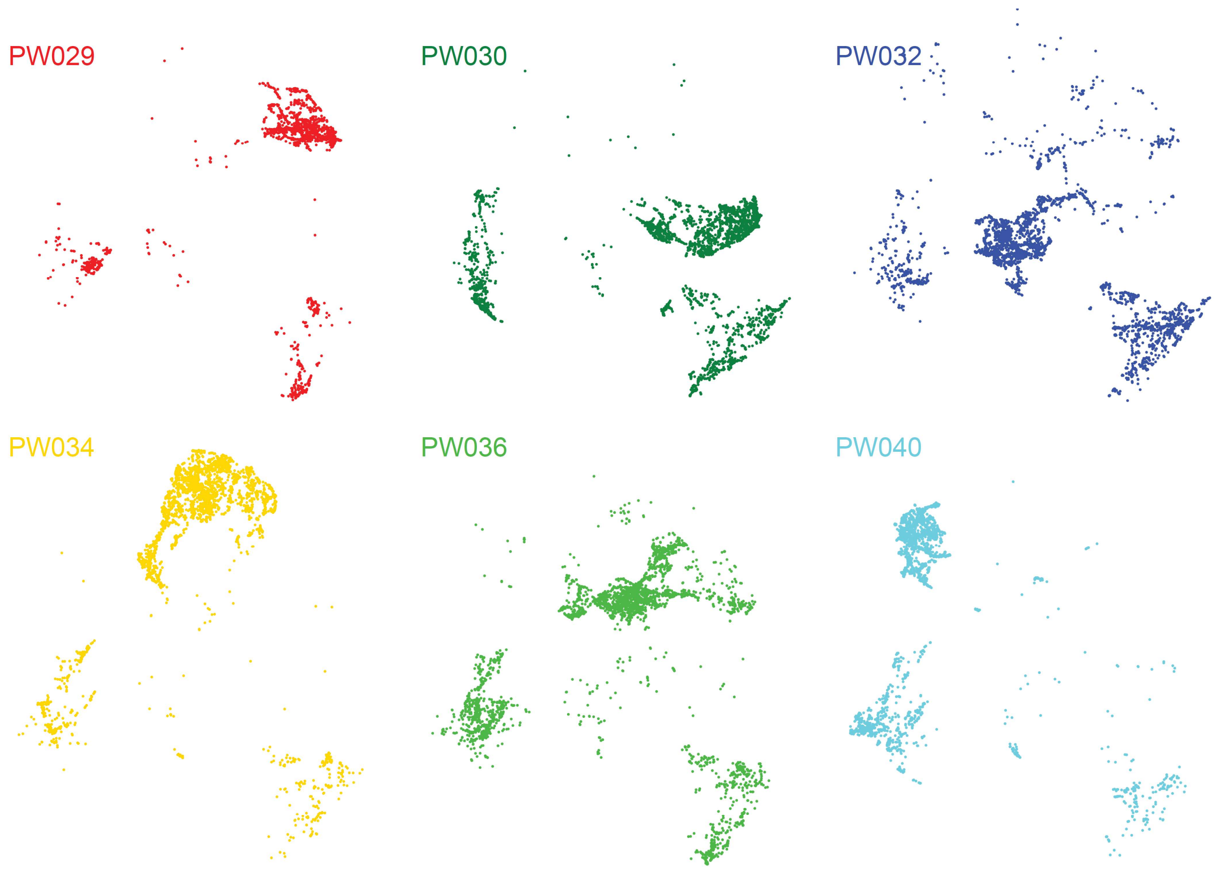

Fig. S8. UMAP embedding in **Fig. 4a** displayed for each sample.

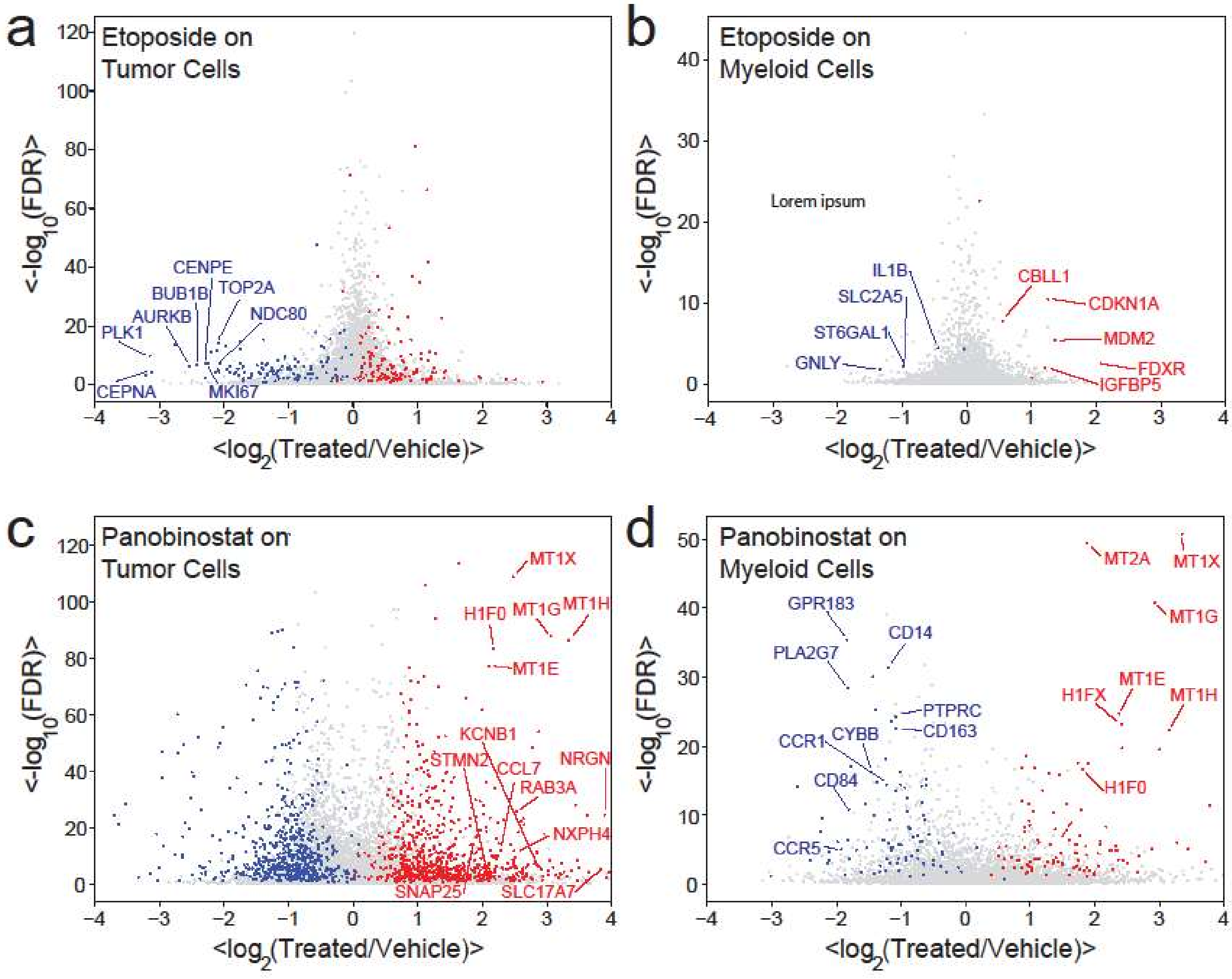

Fig. S9. **a** Volcano plot showing differential expression between etoposide- and vehicle-treated transformed tumor cells average across all patients. Genes highlighted in red and blue have fold-increase or decrease, respectively, greater than two and FDR<0.05 in at least 3/5 patients. A large set of cell cycle control markers are highly downregulated in the etoposide-treated cells. **b** Same as **a** but for the myeloid cells where etoposide has a smaller effect. **c** Same as **a** but for panobinostat-treated transformed tumor cells showing strong induction of metallothioneins and several mature neuronal markers. **d** Same as **c** but for the myeloid cells showing downregulation of the macrophage marker CD163 and strong induction of metallothioneins.

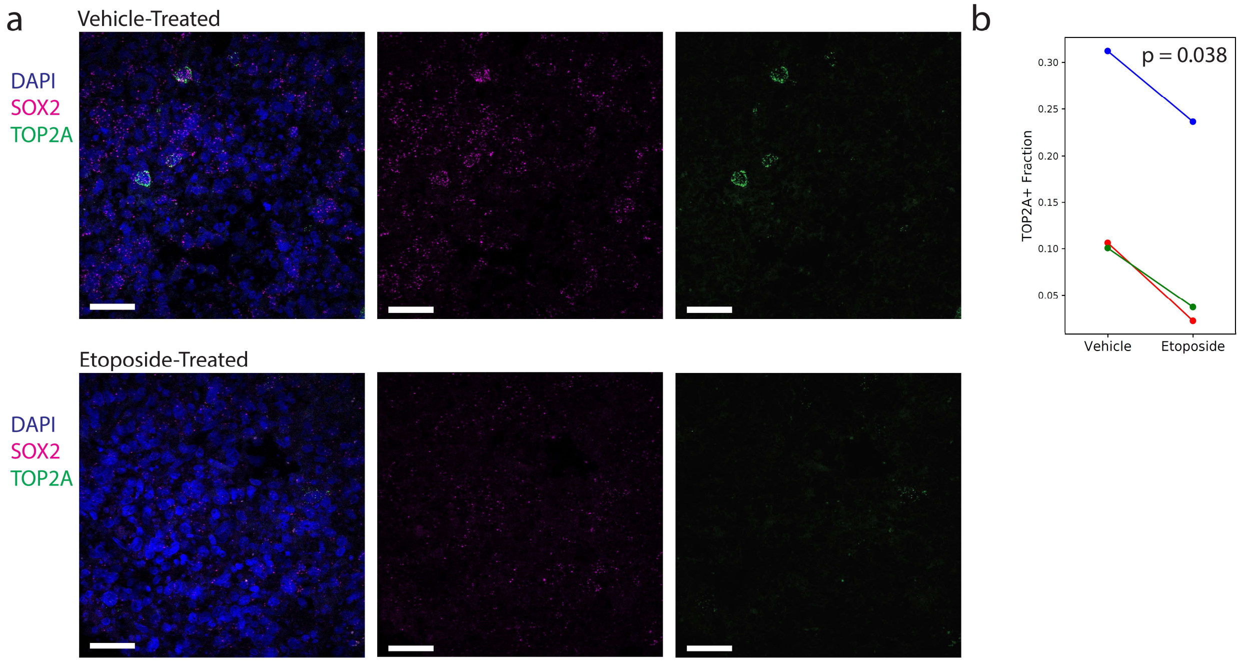

Fig. S10. a Representative images (scale bars = 50 microns) of vehicle- and etoposide-treated slices with nuclear stain (DAPI), SOX2 RNAscope, and TOP2A RNAscope demonstrating a loss of TOP2A+ cells after etoposide treatment. **b** The plot shows the etoposide-mediated decrease in the fraction of TOP2A+ cells based on DAPI segmentation of nuclei for slice cultures from three GBM patients (average fold-decrease = 2.9, p = 0.038 from paired t-test).

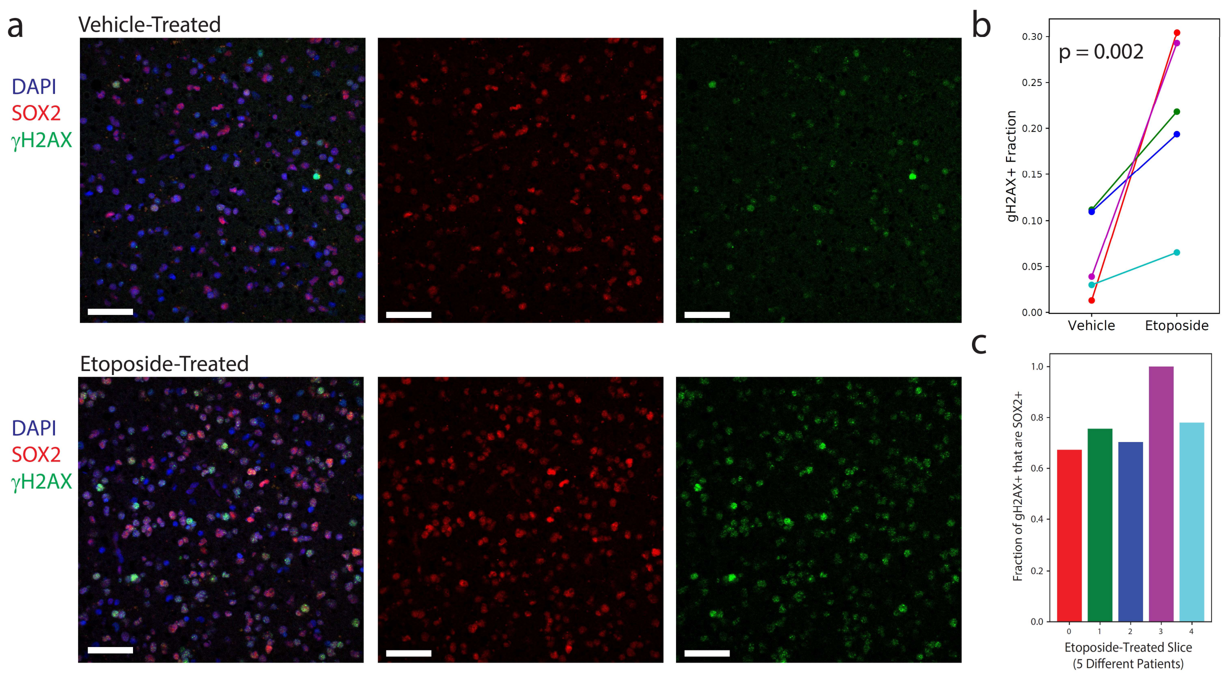

Fig. S11. **a** Representative images (scale bars = 50 microns) of vehicle- and etoposide-treated slices with nuclear stain (DAPI), SOX2 immunofluorescence, and γH2AX immunofluorescence demonstrating an increase in γH2AX+ cells after etoposide treatment. **b** The plot shows the etoposide-mediated increase in the fraction of γH2AX+ cells based on DAPI segmentation of nuclei for slice cultures from five GBM patients (average fold-increase = 7.3, $p = 0.002$ from paired t-test). **c** Bar graph showing the fraction of γH2AX+ cells that are SOX2+ in etoposide-treated slice cultures from five GBM patients (range from ~65-100%).

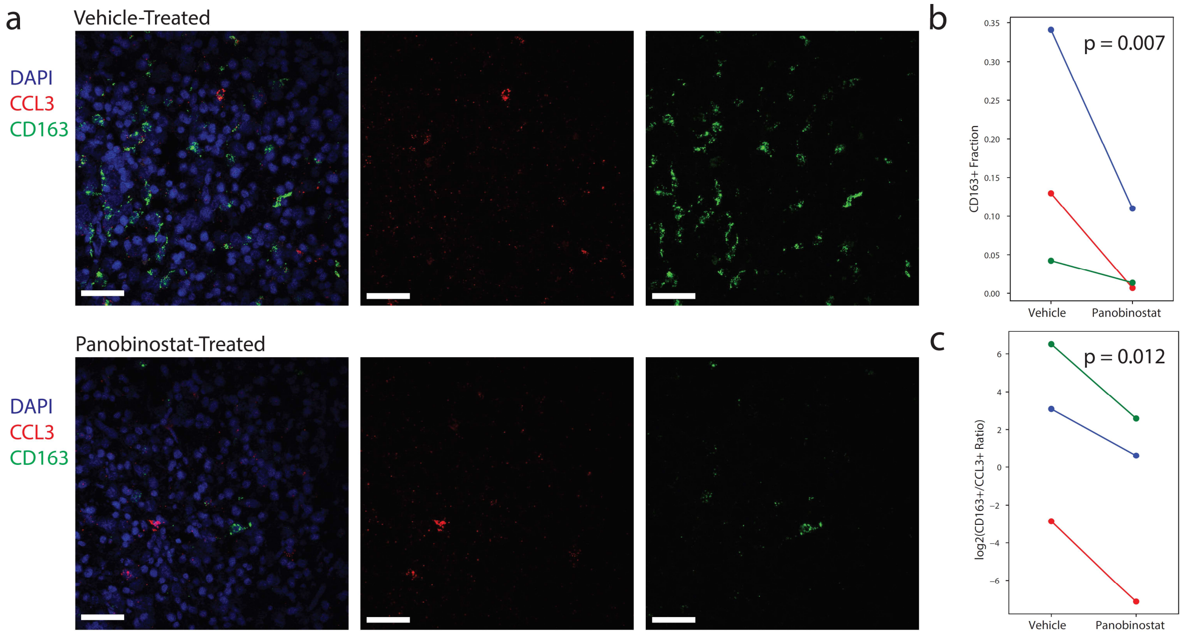

Fig. S12. a Representative images (scale bars = 50 microns) of vehicle- and panobinostat-treated slices with nuclear stain (DAPI), CCL3 RNAscope, and CD163 RNAscope demonstrating a loss of CD163+ cells after panobinostat treatment and a loss of CD163+ cells relative to CCL3+ cells after panobinostat treatment. **b** The plot shows the panobinostat-mediated decrease in the fraction of CD163+ cells based on DAPI segmentation of nuclei for slice cultures from three GBM patients (average fold-decrease = 8.6, $p = 0.007$ from paired t-test). **c** The plot shows the panobinostat-mediated decrease in the fraction of CD163+ cells relative to CCL3+ cells for slice cultures from the same three GBM patients as **b** ($p = 0.012$ from paired t-test).

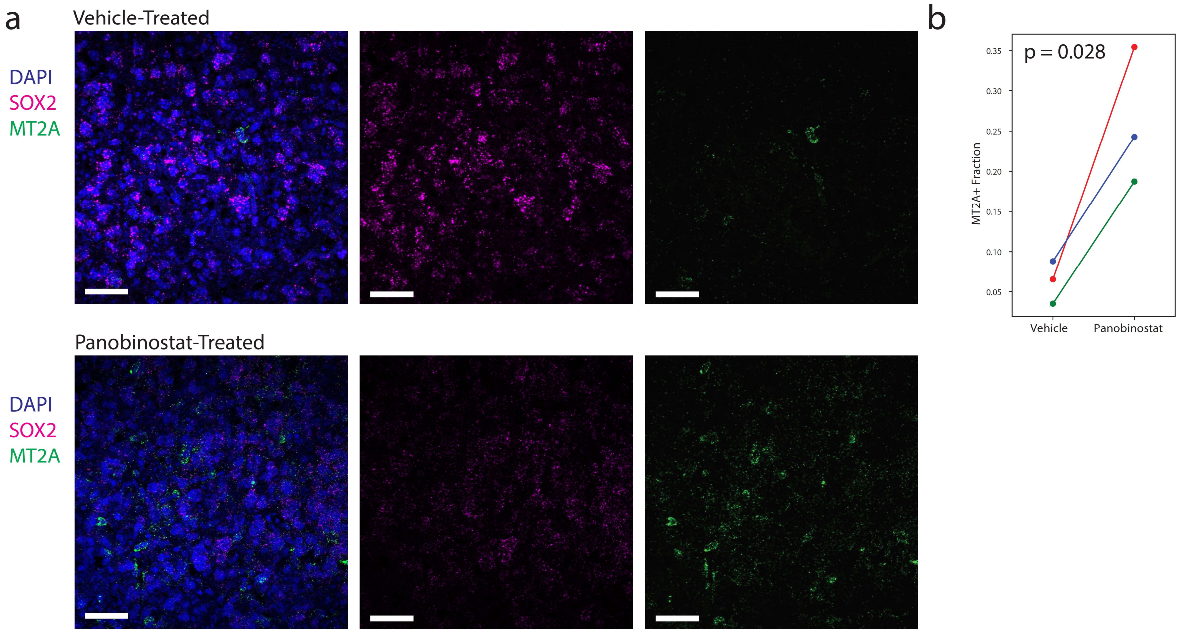

Fig. S13. a Representative images (scale bars = 50 microns) of vehicle- and panobinostat-treated slices with nuclear stain (DAPI), SOX2 RNAscope, and MT2A RNAscope demonstrating an increase in MT2A+ cells after panobinostat treatment. We note that MT2A induction is not limited to SOX2+ cells, consistent with the widespread expression observed by scRNA-seq in panobinostat-treated slice cultures. **b** The plot shows the panobinostat-mediated increase in the fraction of MT2A+ cells based on DAPI segmentation of nuclei for slice cultures from three GBM patients (average fold-increase = 4.4, p = 0.028 from paired t-test).

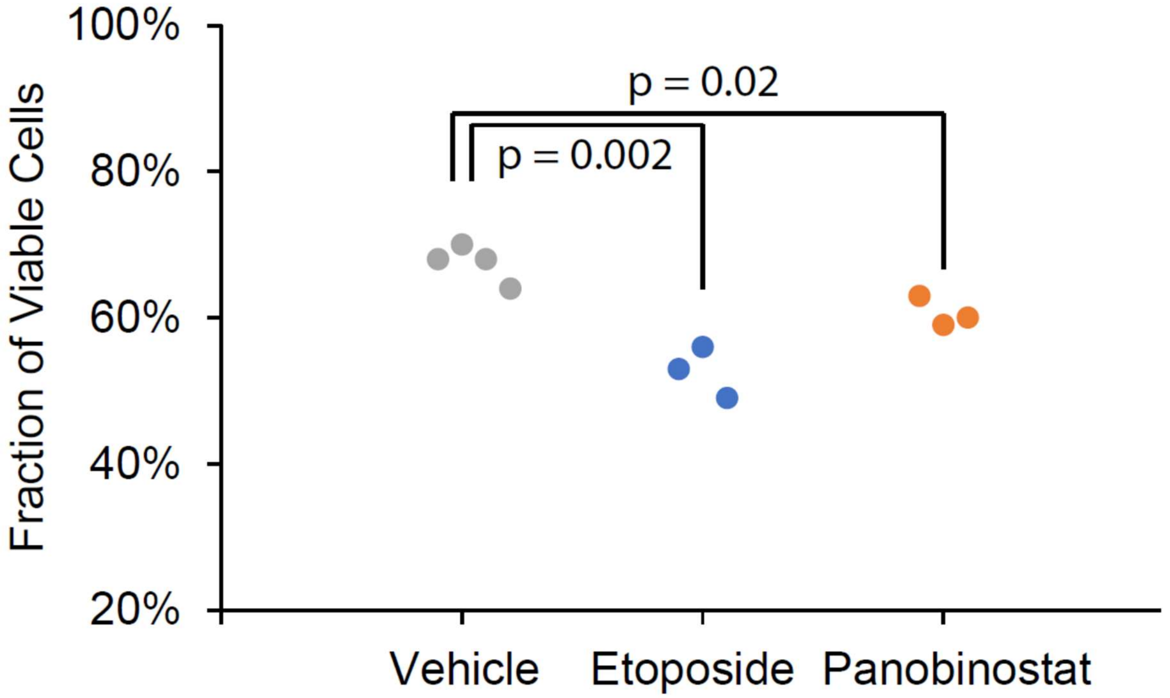

Fig. S14. Fraction of viable cells after slice culture dissociation (each point is a slice culture from TB6393) showing that both sets of drug-treated replicates exhibit significantly lower viability than the vehicle-control slices based on Trypan Blue viability analysis.

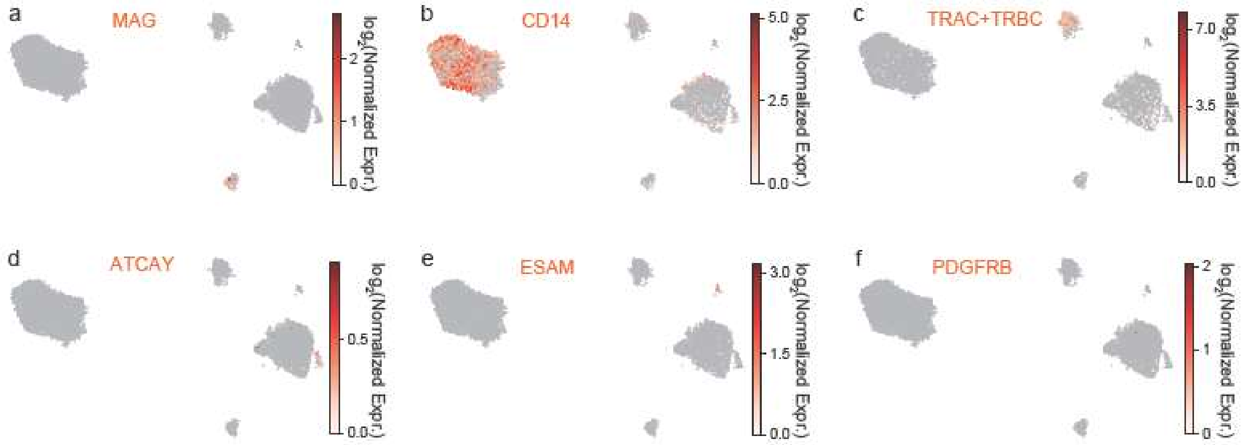

Fig. S15. **a** UMAP embedding of scRNA-seq profiles from ten slices from a single patient (TB6393) described in **Fig. 5** colored by expression of the oligodendrocyte maker MAG. **b** Same as **a** but colored by expression of the myeloid maker CD14. **c** Same as **a** but colored by expression of the T cell receptor constant regions (TRAC, TRBC1, TRBC2). **d** Same as **a** but colored by expression of the expression of the neuronal marker ATCAY. **e** Same as **a** but colored by expression of the endothelial cell marker ESAM. **f** Same as **a** but colored by expression of the pericyte marker PDGFRB.

Supplementary Tables

Sample	Age Range	Sex	Location	Diagnosis	IDH1 Status	EGFR status	Samples	Fig. 4 Samples
PW029	50-59	F	splenial glioma extension into left parietal	Glioblastoma, WHO grade IV	wt	amplified	1 vehicle slice, 1 etoposide slice	1 vehicle slice, 1 etoposide slice
PW030	60-69	M	right parietal	Glioblastoma, WHO grade IV	wt	unamplified	2 vehicle slices, 1 etoposide, 1 panobinostat, 1 ana-12, 1 ispinesib, 1 tazemetostat, and 1 RO492997 slice	2 vehicle slices, 1 etoposide slice, 1 panobinostat slice
PW032	60-69	M	left frontal	Glioblastoma, WHO grade IV	wt	amplified	3 uncultured biopsies, 3 vehicle slices, 1 etoposide slice, 1 panobinostat slice	2 vehicle slices, 1 etoposide slice, 1 panobinostat slice
PW034	60-69	F	left parieto-occipital	Glioblastoma, WHO grade IV	wt	unamplified	2 vehicle slices, 1 etoposide slice, 1 panobinostat slice	2 vehicle slices, 1 etoposide slice, 1 panobinostat slice
PW036	50-59	M	right temporal	Glioblastoma, WHO grade IV	wt	amplified	2 vehicle slices, 1 etoposide slice, 1 panobinostat slice	2 vehicle slices, 1 etoposide slice, 1 panobinostat slice
PW040	60-69	M	right temporal	Glioblastoma, WHO grade IV	wt	amplified	5 vehicle slices, 1 panobinostat slice	2 vehicle slices, 1 panobinostat slice
TB6393	70-79	F	right frontal	Glioblastoma, WHO grade IV (recurrent)	wt	unamplified	4 vehicle slices, 3 etoposide slices, 3 panobinostat slices (**Fig. 5**)	
TB6186	60-69	M	right parietal	Glioblastoma, WHO grade IV	wt	amplified	1 vehicle slice, 1 etoposide slice, 1 panobinostat slice **(Fig. S11-14)**	
TB6193	60-69	M	left frontal	Glioblastoma, WHO grade IV	wt	unamplified	1 vehicle slice, 1 etoposide slice, 1 panobinostat slice **(Fig. S11-12)**	
TB6199	40-49	M	right fronto-parietal	Glioblastoma, WHO grade IV	wt	amplified	1 vehicle slice, 1 etoposide slice, 1 panobinostat slice **(Fig. S11-14)**	
TB6249	80-89	M	N/A	Glioblastoma, WHO grade IV	wt	N/A	1 vehicle slice, 1 etoposide slice, 1 panobinostat slice **(Fig. S13-14)**	
TB6181	30-39	F	N/A	Astrocytoma, WHO grade III	mt	N/A	1 vehicle slice, 1 etoposide slice **(Fig. S14)**	

Table S1: Summary of patients and specimens used for scRNA-seq.

**Drug Name**	**Resource**	**Cat. #**	**Working Concentration**
Etoposide	Tocris Bioscience	1226/100	2.5 µM
Panobinostat(LBH589)	Selleck Chem	S1030	0.2 µM
Ana-12	TOCRIS	4781 - 10 mg	40 nM
Ispinesib	Selleck Chem	S1452	1.8 nM
Tazemetostat(EPZ-6438)	Selleck Chem	S7128	50 µM
RO492997	Selleck Chem	S1575	50 nM

Table S2: Summary of drugs used for these studies.

Target Gene	Species	Probe	Product Name	Catalog number
TOP2A	Homo Sapiens	C1	RNAscope® Probe- Hs-TOP2A	470321
SOX2	Homo Sapiens	C3	RNAscope® Probe-Hs-SOX2-C3	400871-C3
CD163	Homo Sapiens	C1	RNAscope® Probe-Hs-CD163	417061
CCL3	Homo Sapiens	C3	RNAscope® Probe-Hs-CCL3-C3	455331-C3
MT2A	Homo Sapiens	C2	RNAscope® Probe- Hs-MT2A-C2	525411-C2

Table S3: Summary of RNAscope probes used for validation studies from Advanced Cell Diagnostics.

Table S4: Gene score matrix for each scHPF factor in the model shown in **Fig. 4** (attached as a Microsoft Excel sheet, see Additional file 2).
